# Inhibitory Effects of Traditional Herbal Formula Pyungwi-San on Inflammatory Response *In Vitro* and *In Vivo*

**DOI:** 10.1155/2013/630198

**Published:** 2013-03-06

**Authors:** Ji Young Cha, Ji Yun Jung, Jae Yup Jung, Jong Rok Lee, Il Je Cho, Sae Kwang Ku, Sung Hui Byun, Yong-Tae Ahn, Chul Won Lee, Sang Chan Kim, Won G. An

**Affiliations:** ^1^Division of Pharmacology, School of Korean Medicine, Pusan National University, Yangsan 626-870, Republic of Korea; ^2^College of Oriental Medicine, Daegu Haany University, Gyeongsan 712-715, Republic of Korea; ^3^Department of Pharmaceutical Engineering, Daegu Haany University, Gyeongsan 712-715, Republic of Korea; ^4^Institute of Marine BioTechnology, Pusan National University, Busan 609-735, Republic of Korea

## Abstract

Pyungwi-san (PWS) is a traditional basic herbal formula. We investigated the effects of PWS on induction of cyclooxygenase-2 (COX-2), inducible nitric oxide synthase (iNOS), pro-inflammatory cytokines (interleukin-6 (IL-6) and tumor necrosis factor-**α** (TNF-**α**)) and nuclear factor-kappa B (NF-**κ**B) as well as mitogen-activated protein kinases (MAPKs) in lipopolysaccharide-(LPS-) induced Raw 264.7 cells and on paw edema in rats. Treatment with PWS (0.5, 0.75, and 1 mg/mL) resulted in inhibited levels of expression of LPS-induced COX-2, iNOS, NF-**κ**B, and MAPKs as well as production of prostaglandin E_2_ (PGE_2_), nitric oxide (NO), IL-6, and TNF-**α** induced by LPS. Our results demonstrate that PWS possesses anti-inflammatory activities via decreasing production of pro-inflammatory mediators through suppression of the signaling pathways of NF-**κ**B and MAPKs in LPS-induced macrophage cells. More importantly, results of the carrageenan-(CA-) induced paw edema demonstrate an anti-edema effect of PWS. In addition, it is considered that PWS also inhibits the acute edematous inflammations through suppression of mast cell degranulations and inflammatory mediators, including COX-2, iNOS and TNF-**α**. Thus, our findings may provide scientific evidence to explain the anti-inflammatory properties of PWS *in vitro* and *in vivo*.

## 1. Introduction

Inflammation, characterized by the cardinal signs, including swelling, redness, heat, and pain, is a host response to invading pathogens. In the prolonged term, this response can cause progressive damage, contributing to the pathogenesis of various diseases, such as asthma, arthritis, and inflammatory bowel diseases [1]. There are several pro-inflammatory mediators involved during an inflammatory response. Among them, COX-2, iNOS, and cytokines such as IL-6 and TNF-*α* play major roles in the inflammatory processes and are regarded as crucial anti-inflammatory targets [1, 2]. COX-2 and iNOS inducible enzymes play important roles in production of PGE_2_ and NO. Cyclooxygenase (COX) is the enzyme involved in production of prostaglandins such as PGE_2_. At present, there are three known COX isoenzymes: COX-1, COX-2, and COX-3. Among these enzymes, COX-2 is the enzyme responsible for mediating inflammation by production of PGE_2_ induced by LPS [3, 4]. In addition, NO is a weak radical produced from L-arginine, via the enzyme nitric oxide synthase (NOS). NOS exists in three distinct isoforms: constitutively (cNOS), expressed neuronal NOS (NOS 1 or nNOS) and endothelial NOS (NOS 3 or eNOS), or as an inducible isoform (NOS 2 or iNOS), which is capable of high production output of NO during inflammation [5–7]. In addition, COX-2 and iNOS, contributing to production of PGE_2_ and NO, are proteins whose expression is regulated by activation of NF-*κ*B [8]. NF-*κ*B acts as a transcription factor involved in transactivation of a variety of genes associated with regulation of immune and inflammatory responses, cellular proliferation, and tumorigenesis [9].

Mitogen-activated protein kinases (MAPKs) are serine/threonine-specific protein kinases, consisting of three principal family members, including extracellular signal-regulated kinases (ERKs), c-Jun NH_2_-terminal kinases (JNKs), and p38 [10]. MAPKs play important roles in regulation of the inflammatory response by mediators. The signaling pathways of MAPKs can lead to activation of NF-*κ*B and induce expression of pro-inflammatory genes, including interleukins, TNF-*α*, COX-2, and iNOS [11–13]. IL-6 is a pivotal pro-inflammatory cytokine synthesized primarily by macrophages and plays a role in the acute phase response [14]. In addition, IL-6 stimulates the immune response to tissue damage that leads to inflammation [15]. On the other hand, TNF-*α* is a key mediator in an inflammatory reaction that causes innate immune responses by stimulating release of other inflammatory cytokines [8, 16]. In addition, TNF-*α* is a major mediator of carrageenan-induced inflammation and is able to enhance secretion of leukotrienes (product of lipoxygenase action on polyunsaturated fatty acid such as arachidonic acid) and kinins [17]. Thus, this cytokine induces a number of physiological effects, including cytotoxicity, inflammation, and septic shock [18]. 

The general explanation of Pyungwi-san (PWS) comes from *Collected Treatises of (Zhang) Jing-Yue*; The term Pyung (calming) refers toputting in order that which is unbalanced.Wi (stomach) is not a reference to the stomach organ, but to the entire digestive system, which removes obstruction and stagnation by elimination via the intestines [19]. PWS is a traditional basic herbal formula used as a remedy for common gastrointestinal disorders, like gastritis, esophageal reflux, gastric or duodenal ulcers, and acute or chronic enteritis [20, 21]. In addition, this formula is also prescribed for treatment of gynecological disorders such as amenorrhea, premenstrual syndrome, and cervicitis. Several studies have also reported on the use of PWS for treatment of eczema, pertussis, reduced libido in men, erectile dysfunction, and halitosis [19]. In addition, results of different antioxidant tests, including 1, 1-diphenyl-2picryl-hydrazyl (DPPH) radical scavenging, superoxide anion radical scavenging, metal chelating, hydrogen peroxide scavenging, lipid peroxidation protective effect, and scavenging effect of nitric oxide and peroxynitrite have demonstrated the antioxidative activities of PWS [22]. In addition, Seo et al. [23] reported that PWS exhibited anti-inflammatory activity, mainly through inhibition of PGE_2_ production. However, the molecular action and mechanism behind anti-inflammatory activities of PWS have not been elucidated. Therefore, we investigated the effects of PWS on NF-*κ*B and MAPKs signaling pathways and NF-*κ*B-regulated induction of COX-2, iNOS, and pro-inflammatory cytokines in macrophages. In addition, the effects of PWS on the carrageenan-induced acute edematous inflammation were evaluated by histopathology and histomorphometry. In particular, effects on the changes of total skin thicknesses (from epidermis to dermis), infiltrated inflammatory and mast cell numbers, and COX-2-, iNOS-, and TNF-*α*-positive cell numbers were also observed in the present study. These results provide a basis for the molecular mechanism for understanding the effects of PWS on inflammation.

## 2. Materials and Methods

### 2.1. Chemicals and Reagents

Three reference standards, hesperidin, glycyrrhizin, and magnolol were purchased from Wako Inc. (Japan). Purity of the three reference standards was greater than 98%. HPLC grade solution, acetonitrile, methanol, and reagents were purchased from J. T. Baker (USA). Anti-COX-2, anti-TNF-*α*, anti-NF-*κ*B p65, anti-I*κ*B*α*, and anti-lamin A/C antibodies were obtained from Santa Cruz Biotechnology Inc. (Santa Cruz, CA, USA), and ERK1/2, phospho-ERK1/2, JNK, phospho-JNK, p38, and phospho-p38 antibodies were purchased from Cell Signaling Technology, Inc. (Danvers, MA, USA). In addition, peroxidase-conjugated secondary antibody was purchased from Santa Cruz Biotechnology Inc. Anti-iNOS and anti-*β*-actin antibodies were purchased from Calbiochem (San Diego, CA, USA). The enzyme immunoassay kit for PGE_2_ was purchased from R&D Systems (Minneapolis, MN, USA), and the TNF-*α* and IL-6 ELISA kits were obtained from Pierce Endogen (Rockford, IL, USA). The Vectastain Elite ABC kit and peroxidase substrate kit were purchased from Vector Lab. Inc. (CA, USA). The luciferase assay system was purchased from Promega (Madison, CA, USA). 3-(4,5-dimethylthiazol-2-yl)-2, 5-diphenyl tetrazolium bromide (MTT), sulfanilamide, lipopolysaccharide (LPS), carrageenan, dexamethasone, and all other chemicals were purchased from the Sigma Aldrich Chemical Co. (St. Louis, MO, USA).

### 2.2. Preparation of PWS

The PWS prescription (52.5 g) was composed of the following dried herbal medicines: 15 g of Atractylodis Rhizoma (*Atractylodes japonica*), 10.5 g of Citri Pericarpium (*Citrus unshiu*), 7.5 g of Magnoliae Cortex (*Machilus thunbergii*), 7.5 g of Zingiberis Rhizoma Recens (*Zingiber officinale*), 7.5 g of Zizyphi Fructus (*Zizyphus jujuba*), and 4.5 g of Glycyrrhizae Radix (*Glycyrrhiza uralensis*). These herbal medicines were obtained from the School of Korean Medicine, Pusan National University, Korea. The voucher specimens (PNU10-15) have been deposited into the Herbarium of Ducom. The herbal mixture (52.5 g) was extracted with 800 mL of boiling distilled water for 3 h, and filtered through a filter paper (Hyundai Micro number 20). The supernatant was filtered through a 0.2 *μ*m filter (Nalgene, NY, USA), and the filtrate was then lyophilized. The yield of lyophilized PWS extract was 18.8%. The lyophilized powder of PWS was dissolved in distilled water prior to use.

### 2.3. Profiling the Chemical Contents of PWS by UPLC

#### 2.3.1. Chromatography Conditions

The UPLC (Ultra Performance Liquid Chromatography) system (Waters, USA), equipped with a pump Waters ACQUITY^TM^ ultra performance LC system (USA) and a Waters ACQUITY^TM^ photodiode array detector (PDA), was used for analysis. The Empower Data System was used for recording of the output signal of the detector. A Waters ACQUITY^TM^ BEH C_18_ column (1.7 *μ*m, 2.1 × 100) was used for separation. The mobile phase was composed of water and acetonitrile with the gradient elution system at a flow rate of 0.4 mL/min. The injection volume was 2 *μ*L. The detection UV wavelength was set at 254, and 280 nm. The column temperature was set at room temperature.

#### 2.3.2. Preparation of Standard Solutions and Sample

Standard stock solutions of three marker components, hesperidin, glycyrrhizin, and magnolol, were prepared by dissolving at a concentration of 1000 *μ*g/mL in 10 mL of methanol. Working standard solutions were made by diluting the standard stock solution with methanol. Standard stock solutions and working solutions were stored at 4°C. For preparation of sample, the extract of PWS was weighed and dissolved in methanol at a concentration of 10 mg/mL. Before UPLC analysis, the sample preparation was filtered through a 0.22 *μ*m filter.

### 2.4. Cell Culture

Raw 264.7 mouse macrophage cells (American Type Culture Collection) were maintained in Dulbecco's modified Eagle's medium (DMEM; Hyclone, Thermo Scientific Inc., Bremen, Germany) supplemented with 10% heat-inactivated fetal bovine serum (FBS; Sigma, St. Louis, MO, USA), 100 U/mL of penicillin, and 100 *μ*g/mL of streptomycin (Gibco/BRL, Grand Island, NY, USA) at 37°C in a 5% CO_2_ incubator.

### 2.5. MTT Assay for Cell Viability

For determination of cytotoxic concentrations of PWS, Raw 264.7 cells were plated in a 96-well plate at a density of 5 × 10^4^ cells per well. Cells were serum-starved for 16 h, followed by pretreatment with a variety of concentrations of PWS for 1 h, followed by stimulation with 1 *μ*g/mL of LPS. Cells were then incubated for the next 20 h at 37°C, 5% CO_2_ incubator. Following incubation of the cells, viable cells were stained with 3-(4,5-dimethylthiazol-2-yl)-2,5-diphenyltetrazolium bromide (0.5 mg/mL, 4 h). Media were then removed and formazan crystals produced in the wells were dissolved by addition of 200 *μ*L dimethyl sulfoxide. Absorbance was measured at 570 nm using an ELISA microplate reader (Tecan, USA). Cell viability was defined relative to untreated control cells (i.e., viability (% control) = 100 × (absorbance of treated sample)/(absorbance of control)).

### 2.6. PGE_2_, IL-6, and TNF-*α* Assays

Raw 264.7 cells (5 × 10^5^ cells/mL) were preincubated for 16 h. Cells were then pretreated with a variety of concentrations of PWS for 1 h, followed by stimulation with 1 *μ*g/mL of LPS. Following collection of culture supernatants at 20 h after LPS stimulation, enzyme-linked immunosorbent assay (ELISA) was performed according to the manufacturer's protocol for quantification of levels of PGE_2_, IL-6, and TNF-*α* (PGE_2_, R&D Systems, Minneapolis, MN, USA; IL-6 and TNF-*α*, Pierce Biotechnology, Rockford, IL, USA).

### 2.7. Measurement of Nitric Oxide Production

Following pre-incubation of Raw 264.7 cells (5 × 10^5^ cells/mL) for 16 h, cells were pretreated with a variety of concentrations of PWS for 1 h, followed by stimulation with 1 *μ*g/mL of LPS. Cells were then incubated for 20 h at 37°C, 5% CO_2_ incubator, followed by collection of culture supernatants. Nitric oxide was measured by reaction with 100 *μ*L of Griess reagent (1% sulfanilamide and 0.1% N-(1-naphthy)-ethylenediamine dihydrochloride in 5% phosphoric acid; Roche) to 100 *μ*L of culture supernatant for 15 min at room temperature in the dark. Absorbance was determined at 540 nm using an ELISA microplate reader (Tecan, USA). A standard curve was generated in the same fashion using NaNO_2_.

### 2.8. Reporter Constructs, Transfection, Reporter Cell Line, and Luciferase Assay

Raw 264.7 cells transfected with a NF-*κ*B reporter construct were kindly provided by Prof. Myungsoo Joo (Pusan National University, Korea). Transfected cells (5 × 10^5^ cells/mL) were incubated for 16 h with DMEM containing 10% FBS and pretreated with BPTS for 1 h, followed by stimulation with 1 *μ*g/mL LPS for 20 h. After lysis of cells, luciferase activity was determined using the luciferase assay system (Promega, Madison, CA, USA) and a luminometer (Tecan, USA). NF-*κ*B mediated luciferase activity was normalized according to the amounts of proteins in total cell lysates. The BCA protein assay kit (Pierce, Rockford, IL, USA) was used for the determination of the amount of protein.

### 2.9. Western Blot Analysis

Control and PWS-treated Raw 264.7 cells were collected by centrifugation and washed once with phosphate-buffered saline (PBS). Washed cell pellets were resuspended in extraction lysis buffer (50 mM HEPES (pH 7.0), 250 mM NaCl, 5 mM EDTA, 0.1% Nonidet P-40, 1 mM PMSF, 0.5 mM DTT, 5 mM NaF, and 0.5 mM sodium orthovanadate) containing 5 *μ*g/mL each of leupeptin and aprotinin and incubated for 20 min at 4°C. Microcentrifugation was performed for removal of cell debris, followed by rapid freezing of supernatants. Bio-Rad protein assay reagent was used for the determination of protein concentrations, according to the manufacturer's instructions. Thirty micrograms of cellular proteins from treated or untreated cell extracts were separated on 8% SDS-polyacrylamide gels, followed by electroblotting onto nitrocellulose membranes, followed by incubation overnight with blocking solution (5% skim milk) at 4°C, and then with primary antibody for 2 h. Blots were then washed three times with Tween 20/Tris-buffered saline (TTBS), incubated with a 1 : 1000 dilution of horseradish peroxidase-conjugated secondary antibody for 1 h at room temperature, and rewashed three times with TTBS. ECL Western detection reagents (Amersham Bioscience, Piscataway, NJ, USA) were used for development of blots.

### 2.10. Preparation of Nuclear Extracts

Dishes were washed with ice-cold PBS, scraped, and transferred to microtubes. Cells were allowed to swell by addition of lysis buffer (10 mM HEPES (pH 7.9), 10 mM KCl, 1.5 mM MgCl_2_, 1 mM dithiothreitol, 0.2% NP-40, and protease inhibitor cocktail (Roche Diagnostics, Indianapolis, IN, USA)). Samples were incubated for 10 min on ice and centrifuged for 5 min at 4°C. Pellets containing crude nuclei were resuspended in 50 *μ*L of extraction buffer containing 20 mM HEPES (pH 7.9), 1.5 mM MgCl_2_, 1 mM dithiothreitol, 420 mM NaCl, 20% glycerol, and protease inhibitor cocktail, followed by incubation for 30 min on a shaker at 4°C. Samples were centrifuged at 16,000 ×g for 10 min to obtain supernatant containing nuclear extracts.

### 2.11. Carrageenan-Induced Paw Edema

All animal procedures were performed in accordance with the institutional guidelines for care and use of laboratory animals. Sprague-Dawley rats at four weeks of age (male, 80–100 g) were provided from Samtako Co. (Osan, Korea), acclimatized for one week. Animals were maintained with a supply of filtered pathogen-free air, commercial rat chow (Nestle Purina PetCare Korea Ltd., Seoul, Korea) and water *ad libitum*, and maintained at a temperature between 20°C and 23°C with 12 h light and dark cycles and relative humidity of 50%. Rats (*N* = 25) were randomly divided into five groups, and thus each group consisted of five animals. PWS, dissolved in water, was orally administered to rats at the dose of 0.3 and 1.0 g kg^−1^ day^−1^ for 4 consecutive days. Dexamethasone, an anti-inflammatory drug, was used as a positive control. To induce acute phase inflammation in paw, rats were injected subcutaneously into the right hind paw with a 1% solution of carrageenan dissolved in saline (0.1 mL per animal) 1 h after vehicle or PWS treatment. The paw volumes were measured up to 4 h after the injection at intervals of 1 h. The hind paw volume was determined volumetrically by measuring with a plethysmometer (UGO BASILE; Comerio, VA, ITALY).

### 2.12. Histological Examination

#### 2.12.1. Histological Process

The hind paw skins (*dorsum *and *ventrum pedis *skins) were separated and fixed in 10% neutral buffered formalin, then embedded in paraffin, sectioned (3-4 *μ*m), and stained with hematoxylin and eosin (H&E) for general histopathological profiles, toluidine blue for mast cells, or immunohistochemistry for cyclooxygenase-(COX-) 2, inducible nitric oxide synthase (iNOS), and tumor necrosis factor (TNF)-*α*.

#### 2.12.2. Toluidine Blue Staining

After deparaffinization, prepared histological paraffin sections of paw skin were stained with toluidine blue working solution for 2-3 min (1% toluidine blue contains 1% sodium chloride, pH 2.2–2.5) and then dehydrated, cleared, and mounted.

#### 2.12.3. Immunohistochemistry (IHC)

After deparaffinization of prepared histological paraffin sections of paw skin, citrate buffer antigen (epitope) retrieval pretreatment was performed. Briefly, a water bath was preheated with a staining dish containing 10 mM citrate buffer (pH 6.0) until the temperature reached 95–100°C. Slides were then immersed in the staining dish and the lid was placed loosely on the staining dish, followed by incubation for 20 min. The staining dish was placed at room temperature and the slides were allowed to cool for 20 min. After epitope retrieval, sections were immunostained using avidin-biotin complex (ABC) methods for COX-2, iNOS, and TNF-*α*.

#### 2.12.4. Histomorphometry

In order to observe more detailed changes induced by treatment with CA, measurements of the thicknesses of *dorsum pedis *and* ventrum pedis* skin (from epidermis to dermis; keratin layers were excluded) were performed using an automated image analyzer (*i*Solution FL ver. 9.1, IMT *i*-Solution Inc., Canada) under magnification 40 of microscopy (Nikkon, Japan) at prepared skin histological samples as *μ*m/paw, and infiltrated inflammatory cells and mast cells were also counted using an automated image analyzer as cells/mm^2^ of dermis under magnification 200 of microscopy. In addition, the cells occupied by more than 10% of the density of immunoreactivities against COX-2, iNOS and TNF-*α* were regarded as positive immunoreactive. In the current study, the numbers of COX-2-, iNOS-, and TNF-*α*-positive cells among mm^2^ of dermis were measured using a digital image analyzer, respectively. The histopathologist was blinded to the group distribution when this analysis was performed.

### 2.13. Statistical Analysis

Data were expressed as mean ± S.D. Multiple comparison tests were performed for different dose groups. The Levene test was used for examination of variance homogeneity. If the Levene test indicated no significant deviations from variance homogeneity, the obtained data were analyzed using a one way ANOVA test followed by least-significant differences (LSD) multicomparison test, to determine which pairs of the group comparison were significantly different, and independent *t*-test. In case of significant deviations from variance homogeneity being observed on the Levene test, a nonparametric comparison test, Kruskal-Wallis *H* test, was performed. When a significant difference was observed in the Kruskal-Wallis *H* test, the Mann-Whitney *U* (MW) test was performed to determine the specific pairs of group comparison, which are significantly different. SPSS for Windows (Release 14.0 K, SPSS Inc., Chicago, IL, USA) was used in the performance of statistical analyses. Differences were considered significant at *P* < 0.05. In addition, in this study, changes between control and CA were calculated in order to monitor the severities of acute inflammation induced, and the changes between CA and test material treated skin were also calculated in order to help in understanding the efficacy, as follows: percentage changes compared with control (%) = ((data of CA − data of control)/data of control) × 100, percentage changes compared with CA (%) = ((data of test material treated rats − data of CA)/data of CA) × 100.

## 3. Results

### 3.1. Analysis of PWS

**Figure 1:**
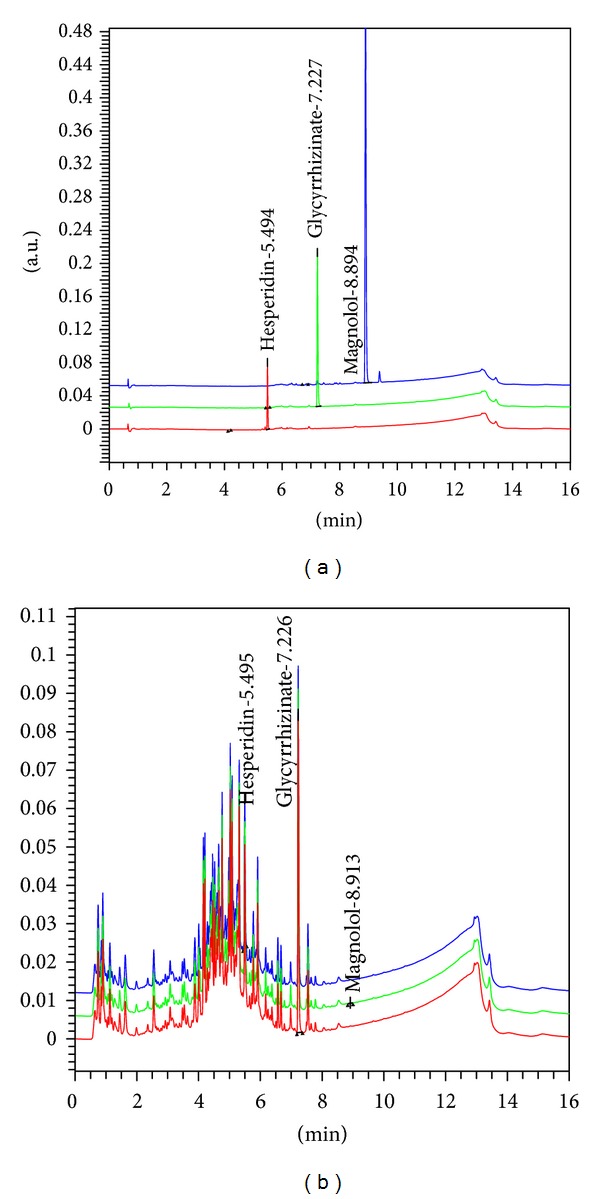
UPLC chromatogram of three marker compounds in PWS. UPLC chromatogram of commercial standard compounds (a). UPLC chromatogram of three marker compounds in PWS (b). The chromatograms were obtained at 254 nm (glycyrrhizin and magnolol) and 280 nm (hesperidin).

Determination of three markers, hesperidin, glycyrrhizin, and magnolol, in PWS was established by the use of the UPLC system. Contents of the three marker components were calculated from the calibration curve of the standards (Table 1 and Figure 1). Validation of the method verified its reliability and stability. Use of the method resulted in successive separation of three marker components in PWS samples.

### 3.2. Inhibitory Effects of PWS on LPS-Induced Production of PGE_2_, NO, IL-6, and TNF-*α* and Transcriptional Activity of NF-*κ*B

**Figure 2:**
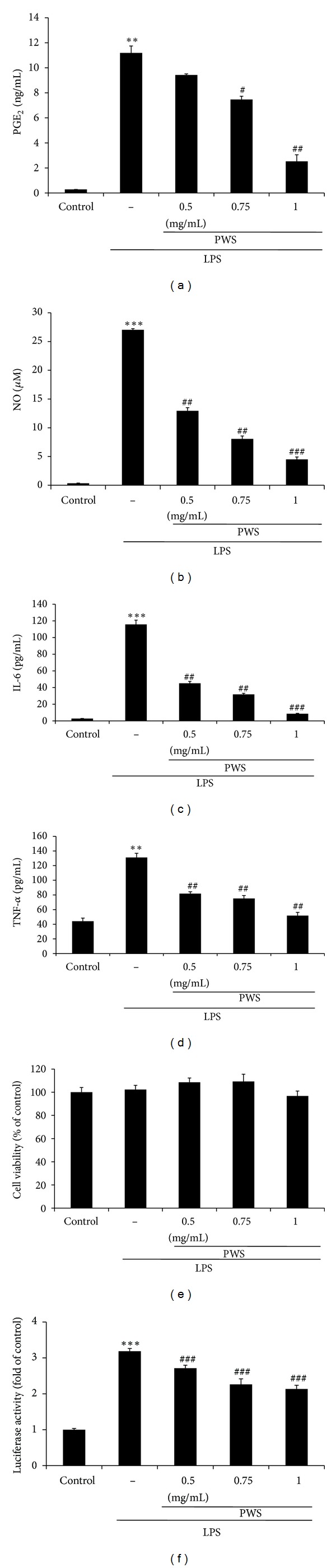
Inhibitory effects of PWS on LPS-induced production of PGE_2_ (a), NO (b), and pro-inflammatory cytokines (IL-6 (c) and TNF-*α* (d)) in Raw 264.7 macrophages. Cells (5 × 10^5^ cells/mL) were treated with various concentrations (0.5, 0.75, and 1 mg/mL) of PWS for 1 h, followed by continuous incubation with LPS (1 *μ*g/mL) for the next 20 h. Concentrations of PGE_2_, NO, and pro-inflammatory cytokines in culture medium were monitored as described in the Materials and Methods Section. The MTT assay was used for measurement of viability of cells exposed to PWS (e). Inhibitory effects of PWS on LPS-induced NF-*κ*B dependent luciferase activity (f). Transfected cells (5 × 10^5^ cells/mL) were incubated for 16 h and pretreated with different concentrations (0.5, 0.75, and 1 mg/mL) of PWS for 1 h, followed by stimulation with 1 *μ*g/mL LPS for 20 h. Following lysis of cells, measurement of luciferase activity was performed using the Promega luciferase assay system and a luminometer. Data represent the mean ± S.D. from three separate experiments. **P < 0.01, ***P < 0.001, significant compared with vehicle-treated control; ^#^ *P* < 0.05, ^##^ *P* < 0.01, ^###^ *P* < 0.001, significant compared with LPS alone.

Different dosages of PWS (0.5, 0.75, and 1 mg/mL) were used for the evaluation of the inhibitory effects of PWS on LPS-induced production of PGE_2_ and NO in Raw 264.7 cells. Compared to control (Lane 1 of Figure 2(a)), treatment with LPS resulted in increased production of PGE_2_ by 39.7-fold (Lane 2 of Figure 2(a)). However, treatment with PWS resulted in reduced production of LPS-induced PGE_2_ in a dose-dependent manner (Lanes 3 to 5, Figure 2(a)). In addition, the same inhibitory effects of PWS on LPS-induced NO production were measured (Figure 2(b)). Compared to control (Lane 1 of Figure 2(b)), treatment with LPS resulted in increased NO production by 84.8-fold (Lane 2 of Figure 2(b)). However, treatment with PWS resulted in significantly reduced LPS-induced NO production in a dose-dependent manner (Lanes 3 to 5, Figure 2(b)). Therefore, PWS exhibited an inhibitory activity for induction of PGE_2_ and NO in macrophages. Because IL-6 and TNF-*α* are important cytokines in the inflammatory reactions, we evaluated the effects of PWS on LPS-inducible production of IL-6 and TNF-*α* by enzyme immunoassay. Compared to control (Lane 1 of Figures 2(c) and 2(d)), treatment with LPS resulted in significantly increased production of IL-6 and TNF-*α* (IL-6, 41.04-fold; TNF-*α*, 2.98-fold) in culture supernatants of Raw 264.7 cells. However, at the concentrations of 0.5, 0.75, and 1 mg/mL, treatment with PWS resulted in significantly inhibited LPS-induced production of IL-6 and TNF-*α* (Lanes 3 to 5 of Figures 2(c) and 2(d)), suggesting that PWS may inhibit expression of these particular genes involved in the inflammation process. Therefore, these results indicate that PWS is a strong inhibitor of induction of IL-6 and TNF-*α*. In addition, the MTT assay was used for examination of possible cytotoxic effects of PWS in Raw 264.7 cells. As shown in Figure 2(e), no change in cell viability was observed according to the different concentrations of PWS treatment, indicating that PWS has no observed cell toxicity. In addition, we also examined the effects of PWS on induction of NF-*κ*B dependent luciferase activity by LPS. Transfected cells were stimulated with 1 *μ*g/mL LPS either in the presence or absence of PWS. Treatment with PWS (0.5, 0.75, or 1 mg/mL) resulted in a significant decrease of LPS-stimulated increases in NF-*κ*B transcriptional activity (Figure 2(f)).

### 3.3. Inhibitory Effects of PWS on LPS-Induced Expression of COX-2 and iNOS

**Figure 3:**
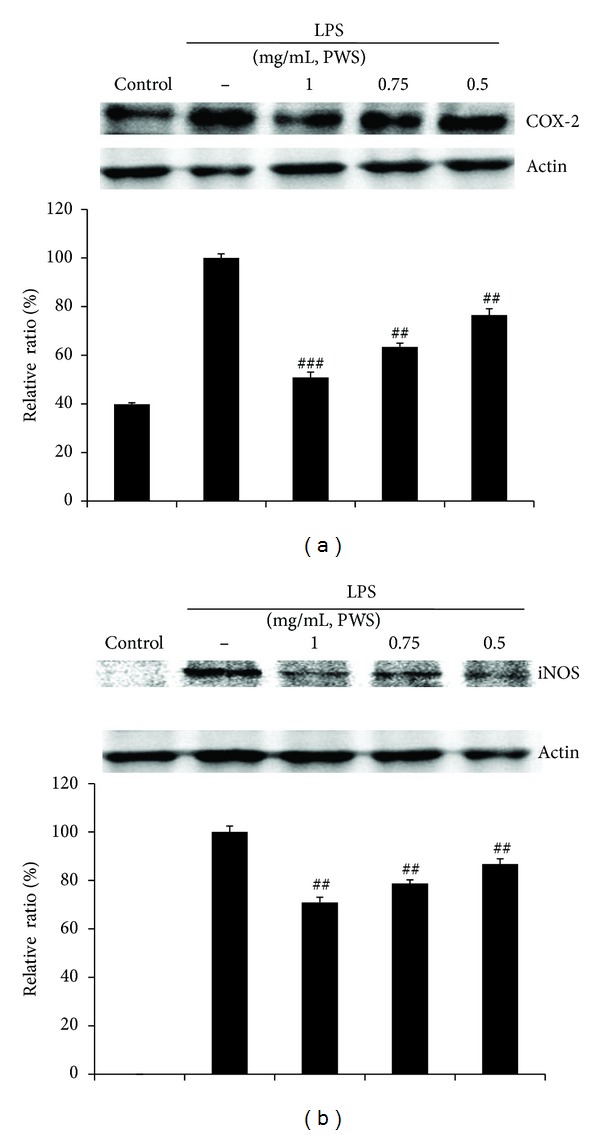
Inhibitory effects of PWS on LPS-induced expression of COX-2 and iNOS. Raw 264.7 cells (5 × 10^5^ cells/mL) were treated with various concentrations (0.5, 0.75, and 1 mg/mL) of PWS for 1 h, followed by continuous incubation with LPS (1 *μ*g/mL) for the next 20 h. Control cells were incubated with vehicle alone. Western blot analysis was performed for determination of protein levels of COX-2 and iNOS. *β*-Actin was used as a loading control. The blots shown are representative of three blots yielding similar results. COX-2 and iNOS versus *β*-actin were measured via densitometry. Data represent the mean ± S.D from three separate experiments. ^##^ *P* < 0.01 and ^###^ *P* < 0.001 indicate significant differences from the LPS-induced group.

Western blot analysis was performed in order to determine whether the inhibitory effects of PWS against PGE_2_ and NO production were related to modulation of COX-2 and iNOS. As shown in Figure 3, outstandingly upregulated levels of COX-2 and iNOSproteinswere observed in response to LPS. Treatment with PWS (0.5, 0.75, or 1 mg/mL) resulted in dose-dependent inhibition of levels of LPS-stimulated COX-2 and iNOSproteins.These findings were consistent with the inhibitory effects of PWS on induction of production of PGE_2_ and NO in LPS-induced Raw 264.7 cells.

### 3.4. Inhibitory Effects of PWS on LPS-Induced Activation of NF-*κ*B

**Figure 4:**
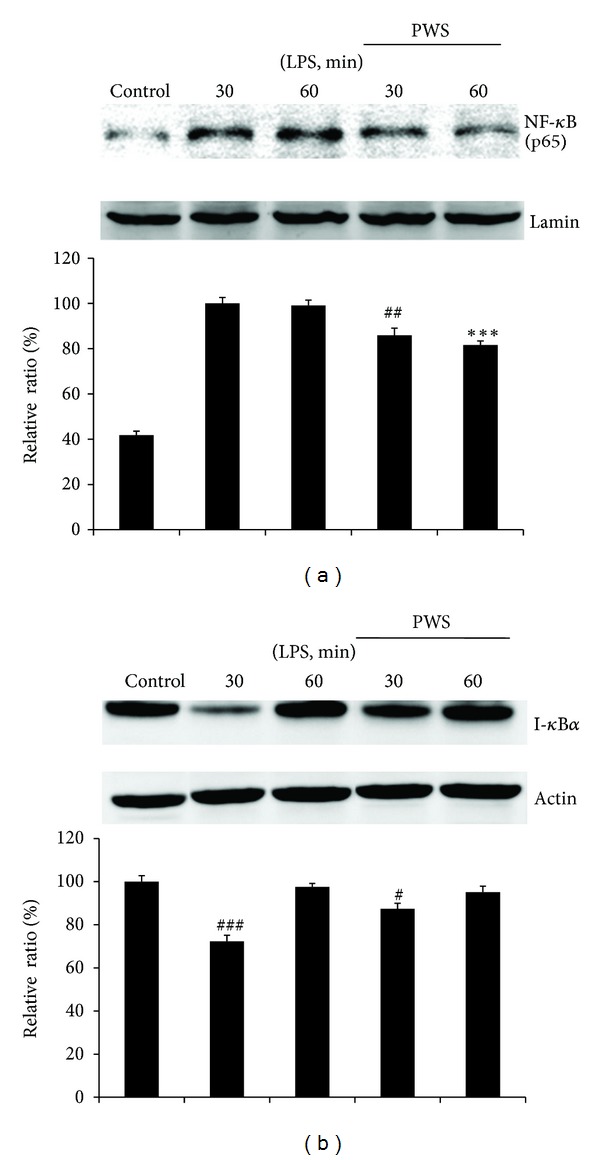
Inhibitory effects of PWS on LPS-induced activation of NF-*κ*B (p65) and degradation of I-*κ*B*α*. Cells (5 × 10^5^ cells/mL) were treated with PWS (0.5 mg/mL) for 1 h, followed by continuous incubation with LPS (1 *μ*g/mL) for 30 and 60 min, respectively. Control cells were incubated with vehicle alone (Lane 1 of (a) and (b)). Nuclear extracts for NF-*κ*B were prepared as described in the methods section. Western blot analysis was performed for determination of protein levels of NF-*κ*B (p65 subunit) (a) and I-*κ*B*α* (b). The blots shown are representative of three blots yielding similar results. NF-*κ*B (p65) versus Lamin A/C (a) and I-*κ*B*α* versus *β*-actin (b) were measured via densitometry. Data represent the mean ± S.D. from three separate experiments. ^##^ *P* < 0.01, significant difference from group (LPS alone, 30 min ((a), Lane 2)); ***P < 0.001, significant difference from group (LPS alone, 60 min ((a), Lane 3)) (a); ^#^ *P* < 0.05 and ^###^ *P* < 0.001, significant differences from control group ((b), Lane 1) (b).

Because the activity of NF-*κ*B is controlled by I-*κ*B*α* [8], regulation of PWS for both NF-*κ*B and I-*κ*B*α* was evaluated by expression of NF-*κ*B and I-*κ*B*α* (Figure 4). As shown in Figure 4(a), outstandingly upregulated levels of NF-*κ*B protein were observed in response to LPS (Lanes 2 and 3). However, treatment with PWS (0.5 mg/mL) resulted in significantly reduced expression of NF-*κ*B protein at 30 min and 1 h after treatment with LPS, respectively (Lanes 4 and 5). In the case of I-*κ*B*α* (Figure 4(b)), its expression was downregulated in response to LPS (Lane 2). However, treatment with PWS resulted in recovered expression of I-*κ*B*α* protein at 30 min after treatment with LPS (Lane 4). Accumulation of newly synthesized I-*κ*B*α* in the cytosol occurs within 60 min [24]. Thus, expression of I-*κ*B*α* proteins with and without PWS treatment at 60 min after LPS treatment was not nearly different (Lanes 3 and 5).

### 3.5. Inhibitory Effects of PWS on LPS-Induced Phosphorylation of MAPKs

**Figure 5:**
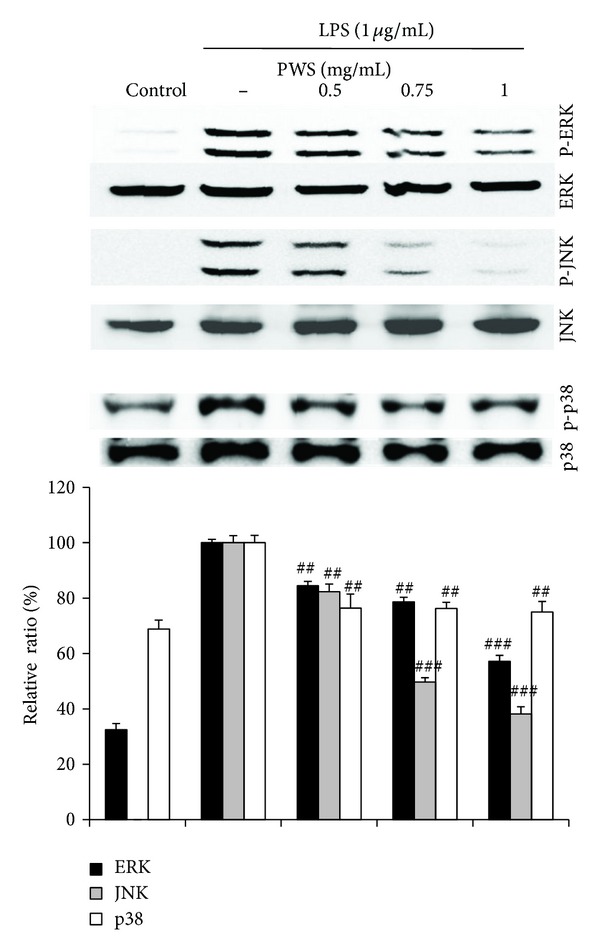
Inhibitory effects ofPWS on phosphorylation of MAPKs (ERK, JNK, and p38) in LPS-induced Raw 264.7 cells. Cells were treated with indicated concentrations of PWS for 1 h, followed by induction with 1 *μ*g/mL LPS for 15 min. Western blot analysis was performed using anti-phosphokinase antibodies for analysis of equal amounts of cell extracts, respectively. Western blot detection of nonphosphorylated kinases was estimated with the protein loading control for each lane. The blots shown are representative of three blots yielding similar results. Data represent the mean ± S.D from three separate experiments. ^##^ *P* < 0.01 and ^###^ *P* < 0.001 indicate significant differences, compared with the LPS-induced group.

To evaluate the molecular target of PWS in the upstream signaling pathway, we examined the effects of PWS on LPS- stimulated phosphorylation of ERK1/2, JNK, and p38 MAPKs in macrophage cells. As shown in Figure 5, treatment with LPS (1 *μ*g/mL) for 15 min resulted in significantly increased phosphorylation of ERK1/2, JNK, and p38 MAPKs. Treatment with PWS (0.5, 0.75, and 1 mg/mL) resulted in significantly decreased phosphorylation of ERK1/2, JNK, and p38 MAPKs.

### 3.6. Inhibitory Effects of PWS on Carrageenan-Induced Rat Hind Paw Edema

**Figure 6:**
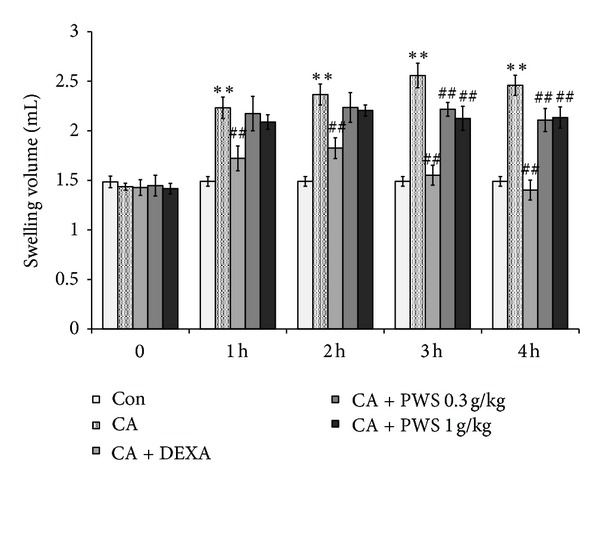
Inhibition of carrageenan-(CA-) induced paw edema by PWS. PWS was administered to rats at an oral dose of 0.3 and 1.0 g/kg/day for four days prior to induction of paw edema. Paw edema was induced by subcutaneous injection of a 1% solution of carrageenan dissolved in saline (0.1 mL per animal) into the right hind paw. The swelling volume of the paw was measured 0–4 h after carrageenan injection. Dexamethasone (DEXA, 1 mg/kg, p.o.) was used as a positive control. Data represent the mean ± S.D of five animals. **P < 0.01, significant compared with control (Con). ^##^ *P* < 0.01, significant compared with carrageenan alone.

In this study, treatment of rats with CA resulted in significantly increased paw swelling in comparison with the control. Pretreatment with dexamethasone (1.0 mg/kg/day, p.o.), a positive control, resulted in a significant decrease in edema formation. Also, treatment with PWS (0.3 and 1 g/kg/day, p.o., for four days) resulted in a significant decrease in paw edema volumes (Figure 6).

### 3.7. Histological Examination

**Figure 7:**
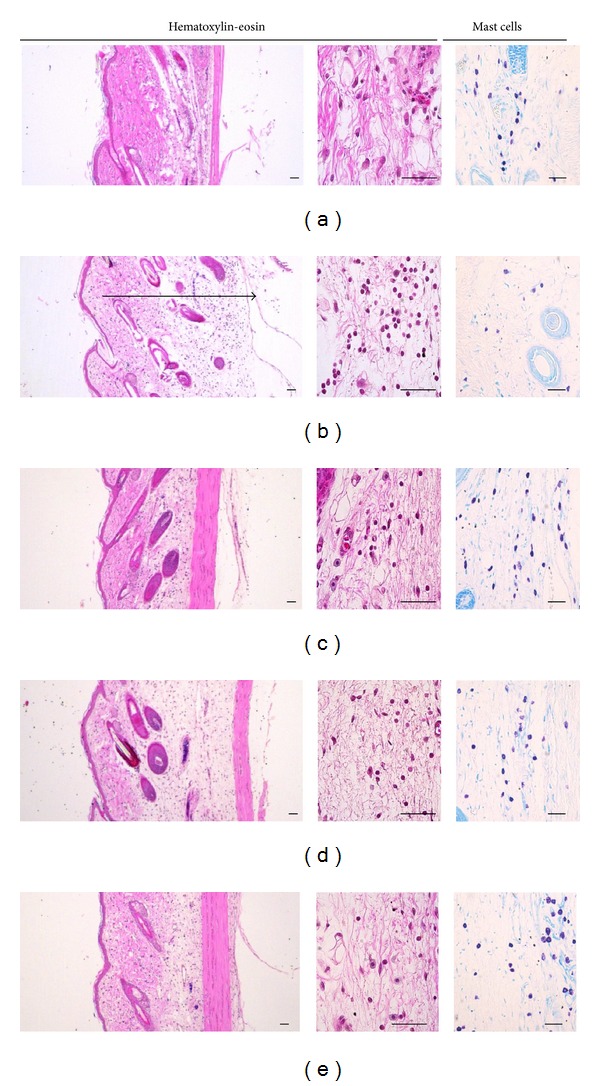
Histological profiles of *dorsum pedis* skin, taken from control and CA-treated rats. Note that marked increases in skin thickness due to edematous changes, with increases of inflammatory cell infiltrations, were detected by CA treatment, as compared with intact control. However, these increases in skin thickness and inflammatory cell infiltrations were effectively inhibited by treatment with dexamethasone and two different dosages of PWS—0.3 and 1.0, respectively. In addition, in this experiment, treatment with dexamethasone and two different dosages of PWS also resulted in favorable preservation of mast cells from the degranulations induced by treatment with CA. (a) control, (b) CA, (c) CA and dexamethasone treated rats, (d) CA and test PWS 0.3 treated rats, (e) CA and test PWS 1.0 treated rats, CA: carrageenan, PWS: *Pyungwi-san*. The arrow indicated total thickness measured. Mast cells were stained with 1% toluidine blue. Scale bars: 40 *μ*m.

**Figure 8:**
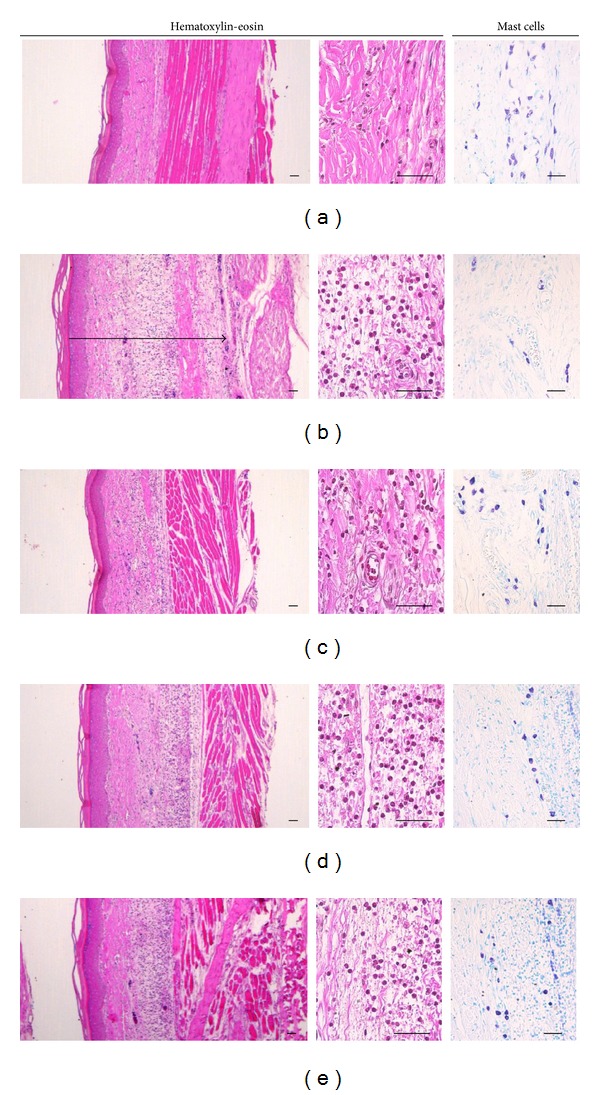
Histological profiles of *ventrum pedis* skin, taken from control and CA-treated rats. Note that marked increases in *ventrum pedis* skin thickness due to edematous changes, with increases in inflammatory cell infiltrations, were detected by CA treatment, as compared with intact control, quite similar to those of *dorsum pedis* skin. However, these increases in skin thickness and inflammatory cell infiltrations were effectively inhibited by treatment with dexamethasone and two different dosages of PWS—0.3 and 1.0, respectively. In addition, in this experiment, treatment with dexamethasone and two different dosages of PWS also resulted in favorable preservation of mast cells from the degranulations induced by treatment with CA. (a) control, (b) CA, (c) CA and dexamethasone treated rats, (d) CA and test PWS 0.3 treated rats, (e) CA and test PWS 1.0 treated rats, CA: carrageenan, PWS: *Pyungwi-san*. The arrow indicated total thickness measured. Mast cells were stained with 1% toluidine blue. Scale bars: 40 *μ*m.

**Figure 9:**
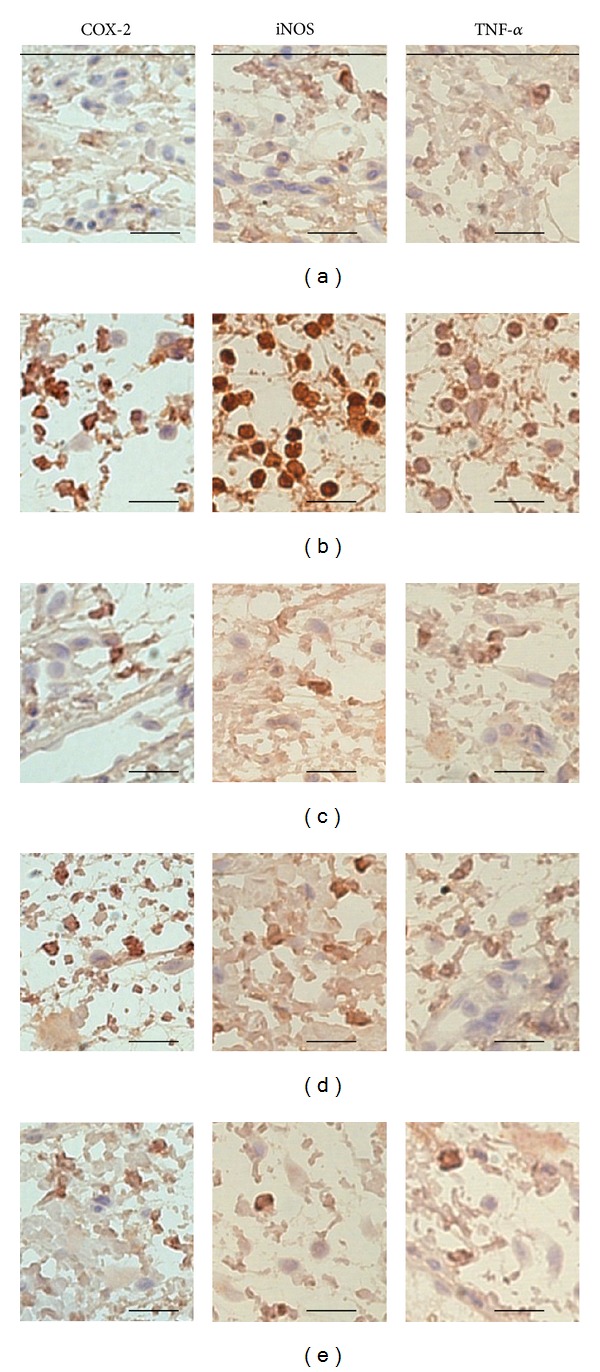
Immunohistochemistrical profiles of *dorsum pedis* skin, taken from control and CA-treated rats. Note that marked increases of COX-2, iNOS, and TNF-*α* positive inflammatory cells were detected in CA rat *dorsum pedis* skin, as compared with control, respectively. However, these increases of COX-2-, iNOS-, and TNF-*α*-immunoreactive cells were effectively inhibited by treatment with dexamethasone and two different dosages of PWS 0.3 and 1.0 g/kg, respectively. (a) control, (b) CA, (c) CA and dexamethasone treated rats, (d) CA and test PWS 0.3 treated rats, (e) CA and test PWS 1.0 treated rats. CA: carrageenan, PWS: *Pyungwi-san*, COX-2: cyclooxygenase-2, iNOS: inducible nitric oxide synthase, TNF-*α*: tumor necrosis factor-*α*. Immunoreactive cells were stained using ABC methods. Scale bars: 80 *μ*m.

**Figure 10:**
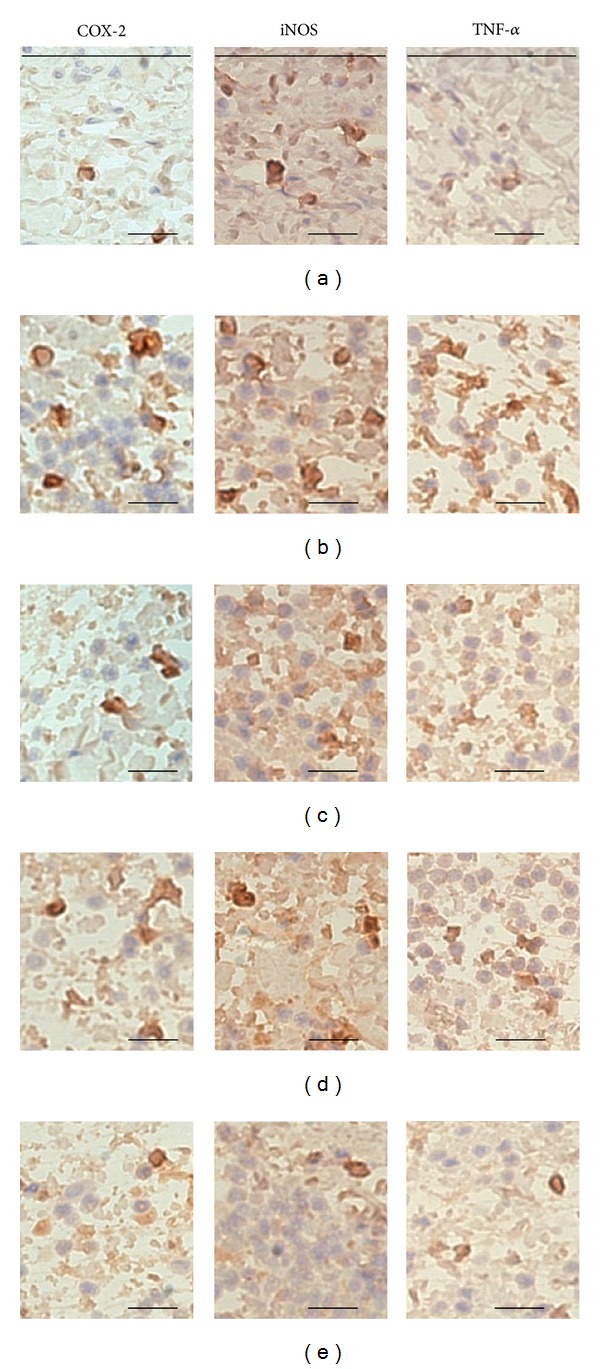
Immunohistochemistrical profiles of *ventrum pedis* skin, taken from control and CA-treated rats. Note that marked increases of COX-2-, iNOS-, and TNF-*α* positive inflammatory cells were detected in CA rat *ventrum pedis* skin, as compared with control, quite similar to those of *dorsum pedis* skin, respectively. However, in this experiment, these increases of COX-2-, iNOS-, and TNF-*α*-immunoreactive cells were effectively inhibited by treatment with dexamethasone and two different dosages of PWS 0.3 and 1.0 g/kg, like those of *dorsum pedis* skin. (a) control, (b) CA, (c) CA and dexamethasone treated rats, (d) CA and test PWS 0.3 treated rats, (e) CA and test PWS 1.0 treated rats, CA: carrageenan, PWS: *Pyungwi-san*, COX-2: cyclooxygenase-2, iNOS: inducible nitric oxide synthase, TNF-*α*: tumor necrosis factor-*α*. Immunoreactive cells were stained using ABC methods. Scale bars: 80 *μ*m.

The effects of PWS observed in this experiment on histological profiles with mast cells of *dorsum *and* ventrum pedis *skin are shown in Figures 7 and 8, respectively. In addition, the representative immunohisto-chemistrical profiles of COX-2-, iNOS-, and TNF-*α*-positive cells in *dorsum *and* ventrum pedis *skin are shown in Figures 9 and 10, respectively. Histomorphometric measurements of *dorsum pedis* and *ventrum pedis* skin are shown in Tables 2 and 3, respectively. Changes in thickness of *dorsum pedis* and *ventrum pedis* skin in CA control were 79.66 and 127.38%, as compared with control, respectively. Changes in thickness of *dorsum pedis* skin in DEXA, PWS 0.3, and 1.0 treated rats were −48.83, −21.66, and −39.86%, and −39.38, −21.37, and −27.32% in *ventrum pedis*, as compared with CA, respectively. In addition, changes in the numbers of infiltrated inflammatory cells on *dorsum pedis* and *ventrum pedis* skin in CA were 622.65 and 5841.67%, as compared with control, respectively. Changes in the numbers of infiltrated inflammatory cells on *dorsum pedis* skin in DEXA, PWS 0.3, and 1.0 treated rats were −75.84, −59.40, and −75.23%, and −63.98, −37.47, and −48.81% in *ventrum pedis* skin, as compared with CA, respectively (Table 2). In addition, changes in the numbers of mast cells on *dorsum pedis* and *ventrum pedis* skin in CA were −65.85 and −74.44%, as compared with control, respectively. Changes in the numbers of mast cells on *dorsum pedis* skin in DEXA, PWS 0.3, and 1.0 treated rats were 126.19, 88.10, and 138.10%, and 189.47, 126.32, and 178.95% in *ventrum pedis* skin, as compared with CA, respectively (Table 3). Changes in the numbers of COX-2-immunoreactive cells on *dorsum pedis* and *ventrum pedis* skin in CA were 150.00 and 409.30%, as compared with control, respectively. Changes in the numbers of COX-2-immunoreactive cells on *dorsum pedis* skin in DEXA, PWS 0.3, and 1.0 treated rats were −58.14, −29.77, and −47.44%, and −64.38, −31.51, and −65.30% in *ventrum pedis* skin, as compared with CA, respectively. In addition, changes in the numbers of iNOS-immunoreactive cells on *dorsum pedis* and *ventrum pedis* skin in CA were 994.12 and 485.34%, as compared with control, respectively. Changes in the numbers of iNOS-immunoreactive cells on *dorsum pedis* skin in DEXA, PWS 0.3, and 1.0 treated rats were −80.29, −54.48, and −75.63%, and −76.29, −51.84, and −73.05% in *ventrum pedis* skin, as compared with CA, respectively. In addition, the numbers of TNF-*α*-immunoreactive cells on *dorsum pedis* and *ventrum pedis* skin in CA were 1351.52 and 1934.48%, as compared with control, respectively. Changes in the numbers of TNF-*α*-immunoreactive cells on *dorsum pedis* skin in DEXA, PWS 0.3, and 1.0 treated rats were −67.22, −30.69, and −66.18%, and −75.59, −38.14, and −74.24% in *ventrum pedis* skin, as compared with CA, respectively (Table 3).

## 4. Discussion

PWS has been used in Korean medicine as a good remedy for common gastrointestinal and gynecological disorders [19–21]. However, there has been little scientific evidence to prove the effects of PWS. Therefore, we evaluated the molecular mechanism of PWS for inflammatory phenomenon.

Inflammation is a response to injury related to chemical or microbiological toxins [1]. LPS, an endotoxin in the outer membrane of Gram-negative bacteria, induces production of PGE_2_, NO, and pro-inflammatory cytokines by activation of transcription factors such as NF-*κ*B and AP-1 in macrophages [8, 25, 26]. Prostaglandins are potent lipid mediators, which have been connected to many pathological processes, including inflammation and cancer [27]. Among them, PGE_2_, a product ofcyclooxygenationby COX-2 of arachidonic acid released from membrane phospholipids, plays major roles in regulation of brain injury and inflammation [28]. This mediator is released by blood vessel walls in response to infection or inflammation that acts on the brain to induce fever. Chemical mediators invoke synthesis of PGE_2_ in monocytes, neutrophils, and endothelial cells at sites of inflammation; therefore, PGE_2_ has been reported to a play a critical role in the inflammatory response [29]. Accordingly, decreases in the level of PGE_2_ have been considered valuable in prediction of the favorable effects of test materials on inflammation [30, 31]. Results of the PGE_2_ assay and Western blotting analysis of COX-2 revealed that treatment with PWS resulted in significantly inhibited induction of PGE_2_ and COX-2 protein by LPS (Figures 2(a) and 3(a)), suggesting that PWS could have a therapeutic effect in treatment of pathogenic heat and pain. Our findings are consistent with Seo's observation that treatment with PWS resulted in significantly inhibited production of PGE_2_ in LPS-induced Raw 264.7 cells [23]. In addition, iNOS causes damages to normal cells through production of a large amount of NO in macrophages treated with LPS [32]. Therefore, in this study, we investigated to determine whether treatment with PWS results in reduced levels of LPS-induced NO production and iNOS protein expression. Our results indicated that treatment with PWS resulted in significantly inhibited induction of NO production and iNOS protein expression by LPS (Figures 2(b) and 3(b)). These results indicate that the therapeutic effect of PWS on some inflammatory symptoms of gastrointestinal and gynecological disorders is due in part to its inhibition of NO production and expression of iNOS protein. In contrast, Seo et al. [23] reported that nitrite levels did not differ significantly in LPS-induced Raw 264.7 cells cultured with or without PWS. Because cells were treated with lower concentrations (50, 100, and 200 *μ*g/mL) of PWS, the inhibitory effects of PWS on induction of NO production are thought to be weak. In addition, pro-inflammatory cytokines (IL-6 and TNF-*α*) are involved in a variety of immunological reactions and interaction with a variety of target cells [14, 16, 33]. Large amounts of these cytokines are released in response to LPS in macrophages via the NF-*κ*B pathway. NF-*κ*B has been shown to play major roles in transmission of inflammatory cytokine signals to the nucleus [14]. In this paper, assessment of the inhibitory effects of PWS on LPS-induced IL-6 and TNF-*α* was performed using the ELISA kit and the data showed that treatment with PWS resulted in significantly inhibited secretion of LPS-induced IL-6 and TNF-*α* (Figures 2(c) and 2(d)). These results suggest that the inhibitory effects of PWS on pro-inflammatory cytokines may have important implications for development of strategies to cure pathological inflammation.

In general, most inflammatory agents mediate their effects through activation of NF-*κ*B; however, anti-inflammatory and chemopreventive agents suppress activation of NF-*κ*B [34]. NF-*κ*B consists of five subunits, p50 (NF-*κ*B_1_), p65 (Rel A), Rel B, c-Rel, and p52 (NF-*κ*B_2_), and acts through formation of homo- or heterodimers, the most abundant of which is a p50/p65 hetero-dimer [35]. This dimer is retained in the cytoplasm by its association with the inhibitory proteins, I-*κ*Bs (I-*κ*B*α*, I-*κ*B*β*, and I-*κ*B*ε*). Upon exposure of cells to LPS, I-*κ*B proteins are phosphorylated and degraded, following NF-*κ*B in translocation to the nucleus. In the nucleus, NF-*κ*B dimers are combined with target DNA elements and activate transcription of the genes encoding for proteins involved in inflammatory response [36, 37]. Our results showed that addition of LPS led to a reduction of I-*κ*B*α* level, whereas treatment with PWS resulted in inhibited nuclear translocation of NF-*κ*B and degradation of I-*κ*B*α* (Figures 4(a) and 4(b)). Kim et al. [38] reported activation of NF-*κ*B at 30 min to 1 h after treatment with LPS. Our findings were consistent with Kim's observation (Figure 4(a)). In addition, pretreatment with PWS resulted in significantly inhibited LPS-induced activation of NF-*κ*B (0.5 mg/mL) at 30 min and 1 h after treatment with LPS (Figure 4(a)). Nuclear translocation of NF-*κ*B is preceded by degradation of I-*κ*B*α* subunit [39]. Because newly synthesized I-*κ*B*α* accumulates in the cytosol within 1 h, the effect of LPS on degradation I-*κ*B*α* is transient [24]. Our data also showed that treatment with LPS caused degradation of I-*κ*B*α*, whereas treatment with PWS resulted in inhibited degradation of I-*κ*B*α*. Consistent with these results, LPS-induced increases in NF-*κ*B dependent luciferase activity were also reduced in response to pretreatment with PWS (Figure 2(f)). These findings suggest that treatment with PWS may inhibit NF-*κ*B activation via suppression of degradation of I-*κ*B*α* and nuclear translocation of NF-*κ*B in LPS-induced Raw 264.7 cells. In addition, involvement of NF-*κ*B in regulation of COX-2 and iNOS expression has been reported [13], and NF-*κ*B has also been reported as a master regulator of LPS-induced release of pro-inflammatory cytokines [39]. Overall, the results also indicate that the inhibitory properties of PWS against NF-*κ*B activation show correlation with inhibition of induction of COX-2, iNOS, IL-6, and TNF-*α*.

Mitogen-activated protein kinases (MAPKs) are protein Ser/Thr kinases that convert extracellular stimuli into a wide range of cellular responses [40, 41]. LPS induction of murine macrophages leading to increased phosphorylation and activation of ERK1/2, JNK, and p38 MAPKs has been reported [42]. Both the ERK and p38 pathways play a role in the upregulation of iNOS and TNF production during macrophage activation [10, 43]. In addition, JNK signaling cascade regulates expression of LPS-stimulated iNOS [44]. In addition, repression of nuclear NF-*κ*B activity and transactivation activity of NF-*κ*B (p65) by specific inhibitors of the ERK and p38 MAP kinase pathways has been reported [45]. In this study, we demonstrated that treatment with PWS resulted in significantly inhibited phosphorylation of MAPKs (ERK, JNK, and p38) (Figure 5). In addition, our results also demonstrated the association of inhibited phosphorylation of MAPKs with NF-*κ*B inactivation. Therefore, these findings suggest that the anti-inflammatory properties of PWS are probably due to inhibition of LPS-stimulated phosphorylation of MAPKs and activation of NF-*κ*B in macrophages.

Because treatment with PWS resulted in significantly reduced induction of COX-2, iNOS, IL-6, and TNF-*α* in Raw 264.7 macrophage cells, our studies were extended in order to determine whether treatment with PWS affected acute inflammation in rats. Carrageenan is a standard phlogistic agent. Carrageenan induces inflammatory responses, including increases in capillary permeability, neutrophil infiltration, release of neutrophil-derived mediators, production of neutrophil-derived free radicals, and paw edema [46–48]. Therefore, local treatment with carrageenan, inducing severe edematous acute inflammations, and a carrageenan-induced hind paw acute edematous inflammation animal model has been widely used as a general efficacy animal model for detection of the anti-inflammatory effects of various drugs [49–51]. Several studies have reported on the induction of peripheral release of NO in the later stage of carrageenan-induced inflammation [52] as well as that of PGE_2_ and pro-inflammatory cytokines [53, 54]. According to our results, administration of PWS resulted in significant inhibition of the degree of swelling of carrageenan-injected paws (Figure 6). These findings suggest that inhibitory phenomena of levels of pro-inflammatory mediators, including PGE_2_, NO, IL-6, and TNF-*α* via suppression of the signaling pathways of NF-*κ*B and MAPKs by PWS in LPS-stimulated Raw 264.7 macrophage cells, may show important mechanisms involved in reduction of swelling of carrageenan-injected paws.

Histopathologically, loosening of connective tissues and inflammatory cell (mainly neutrophils) infiltrations were observed around carrageenan treated sites [55–57]. In the current study, marked increases of infiltrated inflammatory cells with increases of skin thickness on both *dorsum *and *ventrum pedis* were detected by treatment with carrageenan, as compared with intact control rat paw skin. However, these carrageenan-induced acute edematous inflammatory changes were significantly (*P* < 0.01) inhibited by treatment with two different dosages of PWS and dexamethasone (Figures 7 and 8, Table 2). These results are considered as direct evidence indicating that PWS exhibits favorable anti-inflammatory activities. In the current study, the anti-inflammatory activities exerted by PWS 1.0 on carrageenan-induced edematous inflammatory skin changes were comparable to those exerted by dexamethasone. In addition, mast cells are widely distributed throughout the body and play a pivotal role in a variety of allergic and inflammatory disorders. Mast cells have cell surface receptors for IgE and are activated by interaction between antigen-specific IgE bound to the receptors and the relevant antigen [58]. Following activation, mast cells release diverse bioactive substances, including histamine and lipid mediators (prostaglandin D_2_ and leukotrienes), which cause immediate type allergic reaction. Mast cell degranulations are also known to increase at acute [59, 60] as well as chronic stage [61–63] of various inflammatory and allergic diseases. In carrageenan-induced acute inflammations, marked increases of mast cell degranulations in the dermis have been detected, and inhibitions of these mast cell activities, degranulations, have been used as a valuable index for prediction of the efficacy of anti-inflammatory drugs [64–66]. In the current study, treatment with PWS and dexamethasone resulted in marked inhibition of mast cell degranulations and preservation of mast cell numbers in the dermis of carrageenan-induced rats, respectively (Figures 7 and 8, Table 3). These results are considered as direct evidence indicating that PWS exerts anti-inflammatory effects, at least in part, through control of mast cell activation and degranulation. In addition, marked increases of COX-2-, iNOS-, and TNF-*α*-immunoreactive cells were detected in the dermis, mainly in infiltrated inflammatory cells of carrageenan-treated, as compared with control rats. However, these increases of inflammatory mediator positive cells were significantly (*P* < 0.01) decreased by treatment with two different dosages of PWS and also by dexamethasone (Figures 9 and 10, Table 3). Therefore, at least in this experiment, it is considered that PWS inhibits acute inflammation via suppressed expression of inflammatory mediators—COX-2, iNOS, and TNF-*α*.

PWS is an extract from a mixture of six herbal components. Several herbal constituents of PWS have been reported to exert anti-inflammatory effects, as follows. The Citri Pericarpium peel has traditionally been used in Asia as a medicine for improvement of asthmatic and bronchial conditions or blood and cardiac circulation [67]. Choi et al. [67] also reported that hesperidin, a citrus flavonoid, is an inhibitor of hypoxia-inducible factor-1 alpha and cytokines in mast cell-mediated inflammatory responses. In addition, hesperidin has been reported to inhibit COX-2 gene expression in Raw 264.7 cells, suggesting the anti-inflammatory activity of this compound [68]. Jain and Parmar [69] reported on the anti-inflammatory and antioxidative potential of hesperidin in the rat air pouch model of inflammation. Hesperidin has also been reported to have a therapeutic effect on adjuvant arthritis in rats through inhibition of synoviocyte activity [70]. Results from a recent research study demonstrated that glycyrrhizin, a triterpene glycoside present in the roots of licorice (Glycyrrhizae Radix), causes broadly inhibited induction of pro-inflammatory mediators induced by TLR 9 agonist CpG-DNA in Raw 264.7 cells and exerts strong attenuation of inflammatory responses induced by TLR 3 and TLR 4 ligands [71]. In addition, glycyrrhizin has been shown to have anti-inflammatory effects, in part, through inhibition of high mobility group box 1 (HMGB1) phosphorylation and secretion [72]. And, inhibition of interleukin-8 production and NF-*κ*B activity in lung epithelial cells and attenuated development of carrageenan-induced lung injury in mice by treatment with glycyrrhizin have been reported [73, 74]. Finally, magnolol, the active compound of the Magnoliae Cortex, which has anti-inflammatory activities, can induce a reduction in levels of PGE_2_ and leukotrien-B4 in pleural fluid of mice and inhibit formation of thromboxane-B2 [75]. Wang et al. [76] reported that magnolol exerts an anti-inflammatory and analgesic effect in both normal and adrenalectomized mice. They suggested that it may be dependent on reduction of eicosanoid mediator in the inflammatory site. In addition, magnolol was found to have potential anti-inflammatory properties via inhibition of nuclear translocation and phosphorylation of the p65 subunit of NF- *κ*B through I-*κ*B kinase inactivation in U937 cells [77]. In terms of chromatographic analysis, Yamauchi et al. [78] reported that the five main components, including hesperidin, 6-gingerol, honokiol, glycyrrhizin, and magnolol in PWS were determined by ion-pair high-performance liquid chromatography. In addition, Shin et al. [21] reported that the major active ingredients of PWS include liquiritin, glycyrrhizin, and hesperidin. However, results of our study showed that the main markers of PWS included hesperidin, glycyrrhizin, and magnolol (Table 1 and Figure 1). Thus, because marker compounds in herbs can be affected by many factors, including place, collection time, temperature, cultivation environment of the plants, and method of manufacture of the herbal medicine, these analytical results were somewhat different. In this paper, we suggest that the anti-inflammatory activities of PWS on LPS-stimulated macrophages and carrageenan-induced rat hind paw edema are probably due to three compounds, hesperidin, glycyrrhizin, and magnolol.

In conclusion, we demonstrate here that PWS possesses anti-inflammatory activities via decreasing production of pro-inflammatory mediators (PGE_2_, NO, IL-6, and TNF-*α*) through suppression of the signaling pathways of NF-*κ*B and MAPKs in LPS-induced macrophage cells. More importantly, results of the carrageenan-induced rat hind paw edema assay indicated an antiedema effect of PWS. In addition, it is considered that PWS also inhibits the acute edematous inflammations via suppression of mast cell degranulations and inflammatory mediators, including COX-2, iNOS and TNF-*α* because test material dose-dependently inhibited the CA-induced increases of skin thicknesses; mast cell degranulations; and infiltrated inflammatory, COX-2-, iNOS-, and TNF-*α*-positive cells in both the *dorsum* and *ventrum pedis* skin, respectively. These results also support the traditional use of PWS in some inflammatory symptoms of gastrointestinal and gynecological disorders. Thus, our findings may provide scientific evidence to explain the anti-inflammatory properties of PWS *in vitro and in vivo*.

## Figures and Tables

**Table 1:** Contents of three marker compounds of  Pyungwi-san by UPLC (*n* = 3).

Compound	Content (*μ*g/mL)
Hesperidin	46.53 ± 0.76
Glycyrrhizin	46.47 ± 1.76
Magnolol	0.221 ± 0.009

**Table 2:** Changes on the histomorphometrical analysis of hind paw skins.

Index group	Skins			
	*Dorsum pedis* skin		*Ventrum pedis* skin	
	Thickness (epidermis to dermis; *μ*m)	Infiltrated inflammatory cells (cells/mm^2^ of dermis)	Thickness (epidermis to dermis; *μ*m)	Infiltrated inflammatory cells (cells/mm^2^ of dermis)
Control	536.68 ± 45.21	36.20 ± 14.92	304.13 ± 28.88	16.80 ± 8.64
CA	964.18 ± 102.77^a^	261.60 ± 34.59^a^	691.54 ± 47.19^a^	998.20 ± 220.95^d^
Dexamethasone	493.37 ± 59.22^c^	63.20 ± 12.91^bc^	419.19 ± 84.22^ac^	359.60 ± 191.49^de^
PWS 0.3	755.30 ± 81.18^ac^	106.20 ± 16.75^ac^	543.75 ± 63.14^ac^	624.20 ± 82.36^de^
PWS 1.0	579.82 ± 90.37^c^	64.80 ± 12.38^bc^	502.50 ± 25.23^ac^	511.00 ± 86.23^de^

Values are expressed as mean ± S.D. of five rat hind paws.

CA: carrageenan; PWS: *Pyungwi-san*.

^a^ *P* < 0.01 and ^b^ *P* < 0.05 as compared with intact control by LSD test.

^c^ *P* < 0.01 as compared with CA control by LSD test.

^d^ *P* < 0.01 as compared with intact control by MW test.

^e^ *P* < 0.01 as compared with CA by MW test.

**Table 3:** Changes in the numbers of mast cells, COX-2-, iNOS-, and TNF-*α*-immunoreactive cells in hind paw skins.

Cells	Control	CA	Dexamethasone	PWS 0.3 g/kg	PWS 1.0 g/kg
*Dorsum pedis* skin					
Mast cells	49.20 ± 9.42	16.80 ± 6.57^a^	38.00 ± 8.17^bc^	31.60 ± 4.22^ac^	40.00 ± 6.20^c^
COX-2 + cells	17.20 ± 3.11	43.00 ± 9.97^a^	18.00 ± 1.58^c^	30.20 ± 6.76^ac^	22.60 ± 3.78^c^
iNOS + cells	10.20 ± 2.39	111.60 ± 40.34^d^	22.00 ± 3.87^df^	50.80 ± 14.31^df^	27.20 ± 8.17^df^
TNF-*α* + cells	6.60 ± 2.07	95.80 ± 14.70^a^	31.40 ± 10.16^ac^	66.40 ± 11.22^ac^	32.40 ± 12.99^ac^
*Ventrum pedis* skin					
Mast cells	44.60 ± 7.50	11.40 ± 2.30^a^	33.00 ± 7.91^ac^	25.80 ± 6.57^ac^	31.80 ± 4.21^ac^
COX-2 + cells	8.60 ± 1.95	43.80 ± 6.50^a^	15.60 ± 2.30^bc^	30.00 ± 7.45^ac^	15.20 ± 1.92^bc^
iNOS + cells	23.20 ± 6.06	135.80 ± 37.18^d^	32.20 ± 17.60^f^	65.40 ± 18.56^df^	36.60 ± 11.15^ef^
TNF-*α* + cells	5.80 ± 1.64	118.00 ± 28.28^d^	28.80 ± 9.60^df^	74.00 ± 10.89^df^	30.40 ± 2.41^df^

Values are expressed as mean ± S.D. of five rat hind paws, cells/mm^2^ of dermis.

CA: carrageenan; PWS: *Pyungwi-san*; COX-2: cyclooxygenase-2; iNOS: inducible nitric oxide synthase; TNF-*α*: tumor necrosis factor-*α*.

^a^ *P* < 0.01 and ^b^ *P* < 0.05 as compared with intact control by LSD test.

^c^ *P* < 0.01 as compared with CA control by LSD test.

^d^ *P* < 0.01 and ^e^ *P* < 0.05 as compared with intact control by MW test.

^f^ *P* < 0.01 as compared with CA control by MW test.
